# A feasibility study on preoperative carbohydrate loading in older patients undergoing hip fracture surgery

**DOI:** 10.1186/s12877-024-04958-7

**Published:** 2024-05-06

**Authors:** Kai Sing Yap, PS Loh, Yi Xian Foong, Chu Zhen Mok, Terence Ong, Hui Min Khor

**Affiliations:** 1 Department of Anaesthesiology, Faculty of Medicine, Universiti Malaya, 50603 Kuala Lumpur, Malaysia; 2 Department of Dietetics, University of Malaya Medical Centre, Kuala Lumpur, Malaysia; 3 Department of Medicine, Faculty of Medicine, Universiti Malaya, Kuala Lumpur, Malaysia

**Keywords:** Carbohydrate loading, Hip fracture, Elderly

## Abstract

**Background:**

Preoperative carbohydrate loading in Enhanced Recovery After Surgery is an independent predictor of postoperative outcomes. By reducing the impact of surgical stress response, fasting-induced insulin resistance is modulated. As a clear fluid, consuming carbohydrate drink is safe up to 2 h preoperatively. Widely practiced in abdominal surgeries, its implementation in hip fracture surgeries is yet to be recognized. This study aimed to identify the feasibility of preoperative carbohydrate loading in hip fracture surgery and assess its clinical effects.

**Methods:**

This was a randomized controlled, open labelled trial. Patients ≥ 65 years old without diabetes mellitus, has hip fracture were recruited in a tertiary hospital between November 2020 and May 2021. The intervention was carbohydrate loading versus standard preoperative fasting.

**Results:**

Thirty-four ASA I-III patients (carbohydrate loading and control, *n* = 17 each), mean age 78 years (SEM ± 1.5), mean body mass index 23.7 (SEM ± 0.6 kg/m^2^) were recruited. Analysis for feasibility of carbohydrate loading (*n* = 17) demonstrated attrition rate of 29% (*n* = 5). Otherwise, all recruited patients were compliant (100% compliance) with no adverse events reported. There was no significant difference among groups in the postoperative nausea and vomiting, pain score, fatigue level, muscle strength, postoperative infection and length of hospital stay assessed at 24–48 h postoperatively.

**Conclusion:**

The implementation of preoperative carbohydrate loading was found to be feasible preoperatively in hip fracture surgeries but requires careful coordination among multidisciplinary teams. An adequately powered randomized controlled study is needed to examine the full benefits of preoperative carbohydrate loading in this group of patients.

**Trial registration:**

This study was registered in ClinicalTrial.gov (ClinicalTrials.gov identifier: NCT04614181, date of registration: 03/11/2020).

## Introduction

Hip fracture is prevalent and debilitating in the older population. The projected incidence of hip fracture in this region was 5880 in 2018 and expected to increase 3.55-fold to 20,893 cases by 2050 [1]. This number is alarming because the impact on quality of life after hip fracture is significant, with a one-year mortality of 20% while less than 50% regain their pre-fracture functional status [2].

One in two hip fracture patients are malnourished. The lack of nutrition worsens the catabolic state post-trauma and surgery leading to an increased risk of impaired wound healing, loss of muscle mass, muscle strength and postoperative complications [3]. The post-surgical stress response also causes hormonal and metabolic derangement such as insulin resistance and hyperglycaemia [4], which in addition to a prolonged fasting time, contribute negatively towards the length of hospital stay and cost of hip fracture treatment [5, 6]. Hence, in the past decade, perioperative nutrition has been the focus in postoperative recovery and rehabilitation for hip fracture patients [7].

In the Enhanced Recovery After Surgery (ERAS) program, an important part of the preoperative protocol has been the reduction of fasting time and the introduction of carbohydrate loading up to 2 h prior to surgery [8]. This attenuates many conditions mentioned above such as postoperative insulin resistance, reduced nitrogen production, aggravated protein loss and decline in skeletal muscle mass [9, 10]. The additional benefits include an improved overall well-being postoperatively, with reduction of pain, weakness, nausea, and vomiting which will contribute significantly to an early, successful rehabilitation and recovery [9, 11–13].

Despite knowing the role of preoperative carbohydrate loading in many surgical settings, this concept is new in hip fractures. It has not shown clear evidence of benefit in this patient population, nor has it been completely adapted to local practice in our institution, a university-based tertiary hospital that has an average of 200 hip fracture cases annually.

The main objective of this study was to examine the feasibility of implementing preoperative carbohydrate loading in patients undergoing hip surgery. Secondly, we evaluated the effect of preoperative carbohydrate loading among patients undergoing hip fracture surgery.

## Methods

### Study design, setting and participants

This open labelled, randomized controlled study was approved by the Medical Research Ethics Committee of the hospital (Ethics approval number: 2020629-8833) and registered on ClinicalTrial.gov (ClinicalTrials.gov identifier: NCT04614181, date of registration: 03/11/2020). The research was conducted in accordance with the Declaration of Helsinki. All participants provided written informed consent prior to the study.

A computer-generated randomization method was used to allocate participants to each arm (intervention vs. control) with blocks of 2 on a 1:1 ratio basis and kept in an opaque concealed envelope. All patients admitted for hip fracture were screened based on the following inclusion criteria; ASA (American Society of Anaesthesiologists Physical Status Classification System) I-III, patients aged 65 years and above planned for semi-emergency hip surgery (total arthroplasty, hemiarthroplasty, internal fixation). Those undergoing emergency surgery, revision hip surgery, who had peri-prosthetic hip fracture or fracture due to underlying malignancy were not included. Patients with history of diabetes mellitus, intolerance to carbohydrate drinks, gastric reflux, risk of aspiration, dysphagia and had cognitive impairment (i.e., dementia or delirium) which precluded their ability to provide consent independently were also excluded. Eligible patients were approached for consent, recruited, and had their baseline data recorded before the investigator revealed the allocated randomized group. Data collection was performed between December 2020 and June 2021 in a tertiary hospital.

### Intervention

Three servings of a carbohydrate beverage were planned for the intervention group– two drinks on the day before surgery at 4pm and 8pm and the third, to be consumed 2–6 h prior to the scheduled time of surgery as recommended by the ERAS protocol [14]. A single serving of 237 ml contained 53.6 g of carbohydrate. A note on the timing to serve the beverage was placed on the participant’s chart and verbally informed to the staff nurse in-charge. Information on the timing, amount and completion of each drink was recorded. The control group followed conventional fasting guidelines that allowed solid food up to 6 h and clear fluids up to 2 h before surgery. Participants had follow-up visits on the ward by the research team between 24 and 48 h after surgery. All participants received usual postoperative care which included input from the orthopaedic, geriatric medicine and rehabilitation team.

### Outcome measures

After recruitment, participant’s baseline demographic details, medical history, frailty (Clinical Frailty Scale), functional status and independence (Katz Index of Independence in Activities of Daily Living Scale– Katz ADL and Lawton Instrumental Activities of Daily Living Scale– Lawton IADL), and nutritional status (Malnutrition Universal Screening Tool– MUST) were collected [15–18]. These instruments were selected based on timing needed during the assessment to ensure comfort and minimize rest interruption for participants debilitated with hip fracture.

Muscle strength was taken as the mean of three handgrip strength readings in kilograms (kg) on the dominant arm of patients in a standardized position - supine with shoulders adducted and neutrally rotated, elbow flexed to 90°, wrist 15–30° of dorsiflexion and 0–15° of ulnar deviation using an electronic hand dynamometer (CAMRY Model EH101). Muscle mass was the mid-thigh circumference on the non-operated thigh measured perpendicular to the long axis of the thigh at the mid-point between inguinal crease and proximal border of the patella. The reproducibility for pre- and postoperative measurement was ensured by marking the measurement points.

The primary outcomes considered as feasibility parameters included the **recruitment rate** defined as the average number of patients recruited each month over the period of the study between November 2020 to June 2021; **attrition rate** was the percentage of patients who did not fulfil the complete protocol i.e. consumed all three drinks but did not proceed with operation or proceeded with operation without a total of three drinks; **compliance** as the rate of adherence to the intervention in the number of patients who complied and finished consuming the beverages served; **tolerability** of the drink was scored based on a numerical visual analogue scale (VAS) from 1 to 10 (1 = extremely intolerable, 10 = extremely tolerable); **safety** as any number of adverse events and the severity, if any, after consuming the carbohydrate beverage.

Secondary outcomes to determine short-term clinical benefits of preoperative carbohydrate loading incorporated the incidence of postoperative nausea and vomiting, postoperative pain scores at rest and upon a standardized movement (operated leg raise test to 30°) using the numerical VAS scores ranging from 0 = no pain to 10 = extremely painful, fatigue using the numerical VAS with scores 0 = no fatigue to 10 = extreme fatigue within the first 2 days after surgery. The length of hospital stay calculated in days from the end of surgery to discharge, and postoperative complications such as surgical site infection were also recorded.

### Statistical analysis

The sample size of this study was chosen to evaluate the feasibility of implementing preoperative carbohydrate in clinical practice and therefore not adequately powered to determine significance of secondary measures. A previous publication with similar objective had found an attrition rate of 69% and concluded non-feasibility of conducting randomized controlled trials on preoperative carbohydrate loading in hip fracture.^13^ Therefore, we aimed for a lower rate of attrition in this study to demonstrate otherwise in our institutional practice. Using the formula of 1.96 x √(*p* x (1-*p*) / *n*) *p* = attrition rate, n = sample size published from the National Institute for Health Research NIHR Research Design Service RDS London, a sample size of 30 will estimate an attrition rate of 30% to within a 95% confidence interval of ± 16% [19].

Data was analysed with IBM SPSS Statistics 25.0. Univariate associations between groups were performed with independent samples t-test if normally distributed or Mann-Whitney U test if not normally distributed and presented as mean ± SEM/ SD or median ± interquartile range. For multivariate associations, analysis of variance (ANOVA) was used. A *p*-value of < 0.05 was considered statistically significant.

## Results

**Fig. 1:**
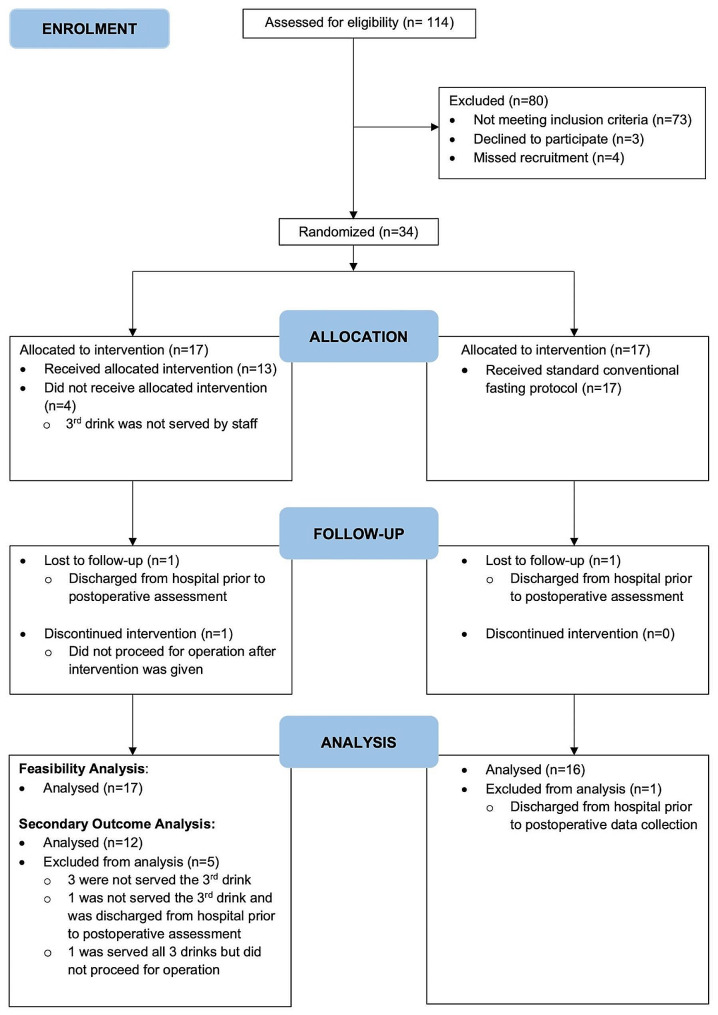
CONSORT (Consolidated Standards of Reporting Trials) Flow Diagram

A total of 114 hip fracture admissions were screened for eligibility. Excluding those who declined to participate and not fulfilling inclusion criteria, a total of 34 participants were recruited from November 2020 to June 2021 and randomized into two groups. All 17 participants in the intervention group were analysed for the feasibility study. A total of 12 patients from the intervention group and 16 patients from the control group were analysed for secondary outcomes. The CONSORT flow diagram is shown in Fig. 1.

The demographic characteristics of both groups (Table 1) were comparable with the mean age of 78 years old, ASA II category as the majority, mean BMI of 23.7 kg/m^2^ and equal gender distribution. Baseline frailty, nutritional status, functional status, handgrip strength and mid-thigh circumference measurement data are shown in Table 2. 80% of the recruited participants had low risk of malnutrition. There was no significant difference in the baseline clinical and functional status between the two groups.

**Table 1:** Characteristics of patients with hip fractures recruited in the randomisation study for pre-operative carbohydrate loading prior to hip fracture surgery

Variables	Categories	All (*N* = 34)	Intervention (*N* = 17)	Control (*N* = 17)	*p*-value
Age (Years)	Mean (SEM)	78 (1.5)	78 (2.2)	77.5 (1.9)	0.77
ASA	I II III	3 (8.8%) 25 (73.5%) 6 (17.6%)	2 (11.8%) 13 (76.5%) 2 (11.8%)	1 (5.9%) 12 (70.6%) 4 (23.5%)	0.60
BMI (kg/m^2^)	Mean (SEM)	23.7 (0.61)	23.1 (0.78)	24.4(0.92)	0.27
Gender	Male Female	16 (47.1%) 18 (52.9%)	7 (41.2%) 10 (58.8%)	9 (52.9%) 8 (47.1%)	0.49
Ethnicity	Malay Chinese Indian	6 (17.6%) 21(61.8%) 7 (20.6%)	3 (17.6%) 12 (70.6%) 2 (11.8%)	3 (17.6%) 9 (52.9%) 5 (29.4%)	0.42
Diagnosis	*Intracapsular*				0.59
	Neck of Femur Fracture	19 (55.9%)	10 (58.8%)	9 (52.9%)	
	*Extracapsular*				
	Intertrochanteric Fracture Subtrochanteric Fracture	14 (41.2%) 1 (2.9%)	7 (41.2%) 0	7 (41.2%) 1 (5.9%)	
Anaesthesia		***N*** ** = 33** ^**a**^	***N*** ** = 16**	***N*** ** = 17**	0.12
	GA only GA + PNB Neuraxial only Neuraxial + PNB	3 (9.1%) 9 (27.3%) 2 (6.1%) 19 (57.6%)	2 (12.5) 6 (37.5%) 2 (12.5%) 6 (37.5%)	1 (5.9%) 3 (17.6%) 0 13 (76.5%)	

ASA, American Society of Anaesthesiologists Physical Status Classification System; BMI, body mass index; GA, general anaesthesia; kg, kilogram; m, metre; PNB, peripheral nerve block; SEM, standard error of mean

^a^One from intervention group did not proceed for operation

**Table 2:** Baseline clinical and functional status

Variables	Categories	All (*N* = 34)	Intervention (*N* = 17)	Control (*N* = 17)	*p*-value
CFS	Median (range) (IQR)	4 (1–7) (2)	4 (1–7) (2)	5 (3–6) (2)	0.11
MUST	Low Risk Medium Risk High Risk	27 (79.4%) 6 (17.6%) 1 (2.9%)	12 (70.6%) 4 (23.5%) 1 (5.9%)	15 (88.2%) 2 (17.6%) 0	0.37
Katz ADL	Median (range) (IQR)	6 (0–6) (0)	6 (1–6) (1)	6 (0–6) (0)	0.73
Lawton IADL	Median (range) (IQR)	6 (0–8) (6)	6 (1–6) (1)	4 (0–6) (0)	0.62
Preoperative handgrip strength (kg)	Mean (SD)	14.2 (6.6)	15.3 (6.8)	13.2 (6.3)	0.37
Preoperative mid-thigh circumference (cm)	Mean (SD)	41.6 (5.5)	40.8 (6.2)	42.3 (4.9)	0.51

CFS, Clinical Frailty Scale; cm, centimetre; IQR, interquartile range; Katz ADL, Katz Index of Independence in Activities of Daily Living; kg, kilogram; Lawton IADL, Lawton Instrumental Activities of Daily Living Scale; MUST, Malnutrition Universal Screening Tool; SD, standard deviation

Results for the primary outcomes showed a recruitment rate of 3–4 patients per month and all patients demonstrated 100% compliance. All who were served the drinks by the staff nurses in charge finished the drinks with no residual leftover. The attrition rate was 29%. Five out of 17 subjects did not complete all three drinks prior to their operation because four were not served the third drink 2 h prior to surgery due to protocol deviation by the staff nurses in charge and one completed three drinks but did not proceed for operation due to a new onset of atrial fibrillation and nosocomial Covid-19 pneumonia. There were no reported adverse events after consuming the carbohydrate beverage. The beverage was very tolerable with a VAS tolerability median score of 10.

An analysis per protocol was conducted for 12 participants from the intervention group and 16 from the control group for the secondary outcomes. Patients excluded from analysis were five in the intervention group who deviated from protocol and one from the control group who was discharged prior to postoperative assessment. Table 3 outlines the postoperative outcomes between the groups. Outcome data was collected at 24–48 h after surgery. There was no significant difference in the VAS for pain at rest (both median 1, *p* = 0.50) or upon movement (median 3 and 4 respectively, *p* = 0.35) for intervention group and control group. Similarly, no significant difference was found when both groups were compared for postoperative fatigue score (intervention group median 2 vs. control group median 4, *p* = 0.07) and length of hospital stay (intervention group median 2.9 days vs. control group median 3.4 days, *p* = 0.25). Postoperative nausea and vomiting were not reported among all participants in both groups.

**Table 3:** Postoperative outcomes comparing intervention and control groups

Postoperative Variables	Categories	All (*N* = 28)	Intervention (*N* = 12)	Control (*N* = 16)	*p*-value
Infection	Frequency (%)	1 (3.5)	0	1 (6.3)	0.38
Pain (at rest)	Median (Range) (IQR)	1 (0–8) (2)	1 (0–8) (2)	1 (0–3) (1)	0.50
Pain (upon movement)	Median (Range) (IQR)	3 (1–9) (2.25)	3 (2–10) (2)	4 (1–8) (1)	0.35
Postoperative Fatigue	Median (Range) (IQR)	3 (1–8) (3)	2 (1–8) (2)	4 (1–7) (3)	0.07
Length of stay	Median (Range) (IQR)	3 (1.2–32.9) (1.65)	2.9 (2.0-3.8) (0.6)	3.4 (1.2–32.9) (5.9)	0.25
Postoperative handgrip strength	Mean (SD) (95% CI)	14.6 (6.3) (12.2–17.1)	15.9 (6.6) (11.7–20.1)	13.7 (6.1) (10.4–16.9)	0.36
Postoperative mid-thigh circumference	Mean (SD) (95% CI)	41.4 (5.6) (39.2–43.6)	41.0 (6.4) (37.6–44.8)	41.7 (5.2) (39.3–44.3)	0.75

CI, confidence interval; IQR, interquartile range; SD, standard deviation

Further analysis found no significant differences in scores of handgrip strength and non-operated mid-thigh circumference among controls and intervention groups pre- and postoperatively. A one way between-group analysis of covariance was conducted to compare the effect of preoperative carbohydrate loading over standard conventional fasting on handgrip strength. After adjusting for pre-intervention handgrip strength, there was no significant difference between the two groups on postoperative handgrip strength (F (1,25) = 0.38, *p* = 0.54, partial eta square = 0.015). Similarly, postoperative mid-thigh circumference did not differ among controls and intervention group (F (1,25) = 2.37, *p* = 0.14, partial eta square = 0.086).

The effect of nutritional status on pre- and postoperative handgrip strength was compared between groups by performing a one way between-group analysis of variance. The scores obtained from the MUST assessment was used to categorize subjects into low risk, medium risk and high risk for malnutrition as a surrogate measure of nutritional status. There was no statistically significant difference in preoperative handgrip strength among the three groups, F (1, 25) = 1.66, *p* = 0.21.

A two way between-groups analysis of variance was conducted to explore the impact of nutritional status, which was again divided to three groups– low, medium and high risk for malnutrition with preoperative carbohydrate loading on postoperative handgrip strength (Table 4). The association between nutritional status and intervention group was not statistically significant, F (2, 23) = 0.276, *p* = 0.60 and the nutritional status had no significant effect on postoperative handgrip strength. The main effect for preoperative carbohydrate loading, F (1, 23) = 2.6, *p* = 0.12 did not reach statistical significance.

**Table 4:** Effect of nutritional status on handgrip strength between groups

		Preoperative Handgrip Strength, Mean (SD)						Postoperative Handgrip Strength, Mean (SD)	
Group		All (*N* = 28)		Intervention (*N* = 12)		Control (*N* = 16)		Intervention (*N* = 12)	Control (*N* = 16)
		*N*	Mean (SD)	*N*	Mean (SD)	*N*	Mean (SD)	Mean (SD)	Mean (SD)
MUST	Low	21	15.8 (6.1)	7	17.9 (7.0)	14	14.8 (5.6)	18.0 (6.7)	15.8 (6.0)
	Medium	6	10.6 (6.9)	4	12.8 (7.3)	2	6.3 (5.1)	13.0 (7.0)	10.8 (6.8)
	High	1	12.5 (-)	1	12.5 (-)	0	-	13.2 (-)	-

MUST, Malnutrition Universal Screening Tool; SD, standard deviation

## Discussion

The idea for preoperative carbohydrate loading stemmed from increasing evidence over the last decades regarding the reduction of preoperative fasting time, starting from the observation that clear fluids given up to 90–180 min before surgery compared to fasting from midnight did not increase the risk of aspiration or residual gastric volume [20]. In fact, studies had found that it is safe to take clear carbohydrate drinks up to 2 h before surgery [21]. Our study demonstrated feasibility for preoperative carbohydrate loading in hip fracture patients as a model of care for clinical practice. A low attrition rate of less than 30%, with good patient compliance and high tolerability clearly supported the feasibility of this intervention. In contrast, many studies although acknowledged preoperative carbohydrate loading as relatively simple, inexpensive and low risk, has found feasibility to conduct trials in clinical practice unattainable when it comes to nutrition in hip fracture [22, 23].

Earlier work has cited heterogenous outcomes in the implementation of preoperative carbohydrate loading as part of the revised fasting protocol. A study in gynae-oncology patients reported good compliance and ease of implementation, citing positive concerted effort from staff and patients [24]. On another note, a few studies found technical and personnel contributory factors which made its implementation challenging [22, 25]. Therefore, learning from their experience, we had conducted a protocol briefing with the nursing team very early at the preparation stage with emphasis on the safe consumption up to 2 h prior to operation.

From the total of 114 hip fracture patients admitted, 34 were successfully recruited into the study. The recruitment rate was 3–4 patients per month, which was significantly lower than our projected target of 12 patients per month. This was largely attributed to the sharp drop in the number of hip fracture admissions during the Covid-19 pandemic. Seventeen participants were randomized into the intervention group and received preoperative carbohydrate loading. We observed 100% compliance from the participants who were served the drinks because of its favourable tolerability. However, a quarter were not served the third drink before surgery, due to a lack of awareness among nursing staff from subsequent working shifts about the protocol to allow clear fluids up to 2 h before surgery. Furthermore, there was also a lack of communication during handover about the correct timing. Awareness and the change in practice for fasting time of clear fluids prior to surgery will probably be the most important hurdle to overcome in instituting preoperative carbohydrate loading in our patient population rather than the carbohydrate beverage itself. Moreover, there were no reported adverse events after taking the carbohydrate beverages as expected from the safety profile of this intervention in other areas of implemented ERAS protocol [26, 27].

A total of 28 out of 34 (82%) patients were analysed to compare the clinical effects of preoperative carbohydrate loading with control. Without adequate power to analyse secondary outcomes, the results of this study are arbitrary findings to encourage future studies that can evaluate significant benefits of carbohydrate loading in hip fractures [28]. None of the participants reported postoperative nausea and vomiting, similar to earlier work [29]. The possibility of antiemetic function has also remained inconclusive [30, 31]. Other findings included lower pain score upon movement, lower fatigue levels postoperatively, and shorter length of hospital stay although they did not reach statistical significance.

A stronger postoperative handgrip strength as a surrogate marker of muscle strength and important indicator of frailty in one of the three pillars of sarcopenia was also observed in the intervention group [32]. Sarcopenia has been shown to be associated with hip fractures leading to poor functional outcome and mortality [33]. Thus, handgrip strength as an objective measurement of overall muscle strength, carries prognosticating role in predicting postoperative recovery success [34]. Although our findings seemed promising, the results were not statistically significant. It is interesting to observe trends of improved wellbeing markers and shorter length of hospital stay among patients who received the preoperative carbohydrate loading that is similar to earlier studies [35]. But of course, ultimately, to find the answer about the clear role of preoperative carbohydrate loading, larger studies must be conducted in the future.

The strength of this study was establishing a well delineated protocol and workflow with a reproducible method to overcome limitations in recruitment and compliance with clear methods for outcome measures. A multidisciplinary team involving the anaesthesiologists, ortho-geriatricians, orthopaedic surgeons, and dietitian was pivotal as it made the planning and execution possible with regular brainstorming for solutions of any arising issues throughout the conduct of the study. In addition, debriefing after cases who deviated from protocol helped to re-establish the understanding and compliance.

Several limitations apply in this study. One of them is the inevitable selection bias that clouds the true effect of nutritional interventions, a limitation that is also commonly cited in many clinical trials for hip fractures with nutrition in mind. The majority of patients were excluded because of diabetes since earlier studies had shown preoperative carbohydrate loading increases postoperative glucose levels [36]. This in turn had been postulated to increase the risk of postoperative infection with the presence of insulin resistance triggered by the surgical and traumatic stress response [37]. However, Talutis et al. found no difference in blood sugar levels for patients with diabetes who were given or not given preoperative carbohydrate drinks [38]. Such a finding may cause a paradigm shift in the preoperative management, a major component in ERAS protocols for diabetic patients in the future. On the same token, there was no blood glucose monitoring in our non-diabetic study population and hence, precluded analysis of trends in blood glucose levels after consumption of the carbohydrate beverage. In some cases, the time interval between serving the third drink and operation was longer than 2 h due to changes in the operation schedule or delays.

Another limitation is the exclusion of hip fracture patients with poor cognitive function. Those patients would have benefitted the most from preoperative carbohydrate loading especially when malnutrition was likely more prevalent in this group of patients. Instead, feasibility was demonstrated in a cohort who was cognitively intact and likely to be compliant with medical advice anyway. Because of this, the validity of the demonstrated result cannot be extrapolated to the general population of hip fracture patients. Although, a larger sample size will increase the power of future studies and add to the literature in this topic, our study served its objective as a pilot for a future framework in this practice,

## Conclusion

In conclusion, our study showed novel insights and evidence of feasibility in introducing a change in fasting protocol for hip fracture patients by incorporating carbohydrate drinks on the day before and up to 2 h prior to surgery. This can be done with reinforcement on the importance and cooperation on a multidisciplinary level, careful coordination and timing involving nursing care, explanation and disclosure to patients and their caregivers to ensure its safe use and success in future research work.

## Data Availability

The datasets used and analysed during the current study are available from the corresponding author on reasonable request.
